# Measuring nurses’ perception of work environment: a scoping review of questionnaires

**DOI:** 10.1186/s12912-017-0256-9

**Published:** 2017-11-21

**Authors:** Rebecka Maria Norman, Ingeborg Strømseng Sjetne

**Affiliations:** 1 Norwegian Institute of Public Health (FHI), PO Box 4404 Nydalen, N-0403 Oslo, Norway; 2 University of Oslo, Faculty of Medicine, Institute of Health and Society, Department of Health Management and Health Economics, PO Box 1130 Blindern, N-0318 Oslo, Norway

**Keywords:** Questionnaires, Work environment, Review, Nurses, Care workers

## Abstract

**Background:**

Nurses’ work environment has been shown to be associated with quality of care and organizational outcomes. In order to monitor the work environment, it is useful for all stakeholders to know the questionnaires that assess or evaluate conditions for delivering nursing care. The aim of this article is: to review the literature for assessed survey questionnaires that measure nurses’ perception of their work environment, make a brief assessment, and map the content domains included in a selection of questionnaires.

**Methods:**

The search included electronic databases of internationally published literature, international websites, and hand searches of reference lists. Eligible papers describing a questionnaire had to be; a) suitable for nurses working in direct care in general hospitals, nursing homes or home healthcare settings; and b) constructed to measure work environment characteristics that are amenable to change and related to patient and organizational outcomes; and c) presented along with an assessment of their measurement properties.

**Results:**

The search yielded 5077 unique articles. For the final synthesis, 65 articles met inclusion criteria, consisting of 34 questionnaires measuring nursing work environments in different settings. Most of the questionnaires that we found were developed, and tested, for registered nurses in a general hospital setting. Six questionnaires were developed specifically for use in nursing home settings and one for home healthcare. The content domains covered by the questionnaires were both overlapping and unique and the terminology in use was inconsistent. The most common content domains in the work environment questionnaires were supportive managers, collaborative relationships with peers, busyness, professional practice and autonomy.

**Conclusions:**

The findings from this review enhance the understanding of how “work environment” can be measured by an overview of existing questionnaires and domains. Our results indicate that there are very many work environment questionnaires with varying content.

**Electronic supplementary material:**

The online version of this article (10.1186/s12912-017-0256-9) contains supplementary material, which is available to authorized users.

## Background

The work environment of nurses and its associations with quality of care is an area of research that has gained attention in recent decades [1]. A widely used approach in such studies is to describe the services from the bedside perspective, by surveying the employees’ perceptions of the characteristics of their daily work [2]. Although the results are inconclusive, studies on the topic support assumptions about associations between nurses’ work environments and patient outcomes, as well as associations with organizational outcomes such as turnover and retention [3–6]. In a review of studies exploring the relationship between work environment and direct measures of patient outcomes [7], ten out of eleven retrieved studies were North American, and most were conducted in acute general hospital settings.

There is a trend in western healthcare systems to strengthen the activities in non-hospital settings, moving healthcare services from hospital settings to long-term care sectors such as nursing homes. According to Buchan & Aiken [8], the general shortage of nurses is partly the result of unfavourable working conditions. Compared to acute care settings, the long-term care sector faces additional strain due to an ageing and shrinking workforce, a perceived lack of status, a relatively high proportion of low-qualified care workers [9], and high turnover among direct care nurses [10].

As a consequence of this, studies of nurses’ work environments, and the quality of the service they deliver, should not be limited to acute care hospitals settings [7]. The need for a broad review of survey questionnaires occurred in the preparation of a survey of nurses’ perception of their work environments and its associations with quality of care in long-term care settings.

### Nursing work environment

Researchers refer to the work environment as, for example: working conditions, practice environment and job characteristics. In this review, we used Lake and Friese’s definition of the nursing work environment: “characteristics of a work setting that facilitate or limit nursing practice ([11] p.2)”.

A literature review conducted by Bae [7] synthesized various work conditions and their respective associations with patient outcomes. The work conditions were grouped in 10 concepts; autonomy, philosophy emphasizing quality of clinical care, nurse participation, supportive managers, collaborative relationships with physicians, collaborative relationships with peers, staffing, decentralization, patient-centred climate, and busyness. Bae’s synthesis indicated that there is some degree of convergence in the topics. All studies were conducted in acute hospital settings and seven out of eleven studies included in that review used a version of the Nursing Work Index (NWI) for data collection. The NWI is a frequently used questionnaire for measuring nurses’ work environments. It was first developed in the USA for hospital registered nurses in 1989 [12], and there are several versions adapted and revised for different settings and different contexts [13–16]. It has been pointed out that the instruments’ properties are unstable [17, 18]. This is acknowledged by the authors of the NWI, who later developed the Essentials of Magnetism (EOM) [19] process measurement tool and subsequently its revised version EOMII [20, 21]. The EOM tool was developed using the 14 Forces of Magnetism [22] as a framework, together with an extensive participant observation and a qualitative interview study, making the tool reflect a more contemporary nursing practice and the practice environments [19].

Our overall goal was to find questionnaires to measure the work environment in long-term care but in the process of conducting the review, we expanded the criteria to include questionnaires that were used in acute care settings, because these questionnaires contain domains of interest that are also applicable to long-term care nursing work environments. We believe that our review is of interest and useful to stakeholders in other areas of nursing practice. In addition, when choosing topics for a questionnaire, it is necessary to prioritize in order to balance the response burden and information needs. The questionnaires identified in the review provided an excellent opportunity to map the work environment domains that were prioritized by a number of authors.

The research questions guiding this study were:

1. Which assessed survey questionnaires measuring nurses’ perception of the work environment can be found in the literature?
2. What are the content domains included in the questionnaires we found?

The description of work environment questionnaires of interest referred to in research question 1 is presented in more detailed under Screening – Inclusion and Exclusion.

## Method

The review is based on the framework for scoping studies outlined by Arksey and O’Malley [23], further enhanced by Levac et al. [24], Khalil et al. [23, 25], Daudt et al. [26]. They proposed that a scoping review should include an iterative five-stage process, further described below. The two authors conducting the present study have expert familiarity with the field, as nurses with experience from different healthcare settings and questionnaire development and assessment.

### Search strategy

A literature search strategy was designed with a basis in research question 1, and criteria described under Screening – Inclusion and Exclusion. The initial source was electronic databases, limited to articles published in peer-reviewed journals in the English or Scandinavian languages. The search was conducted with support from a research librarian. A test-search was first executed in order to identify relevant keywords representing the study topics. An extensive search was performed in October 2015, and updated in December 2016. The following databases were searched: Embase (1974-) Ovid MEDLINE(R) In-Process & Other Non-Indexed Citations, Ovid MEDLINE(R) Daily, Ovid MEDLINE(R) and Ovid OLDMEDLINE(R) (1946 -); PsycINFO (1806-); CINAHL and SweMed + .

We used the keywords and searched in title, index terms and author’s keywords. Several keywords in different combinations, endings, spelling, grammatical forms and synonyms were included in the extensive search. The search strategy was tailored to the best possible fit for each database. We provide the strategy used to search MEDLINE as an example (Table 1). The complete list of search terms can be found in Additional file 1.

**Table 1:** Keywords used to search MEDLINE

Work environment and outcomes
occupational health, occupational safety, employee health, employee safety or occupational injury, working conditions, practice environment, work environment, workload, overwork, work stressor, nurse-patient-ratio, missed or omitted or rationing, nursing left undone or care left undone, work schedule tolerance, workday shifts, work shift, rotating shift, workday shift, work schedule, work rest cycle, personnel turnover, employee turnover, turnover or intention-to-leave, vacancy, personnel staffing and scheduling, work scheduling, staffing, manpower, burnout, professional, occupational stress, burnout, exhaustion, distress, occupational stress, absenteeism, sick leave, sick rate, sick day, illness day, jobwork-, employee-, career satisfaction, employee grievances, personnel-, work-, staff-, nursing grievance, job dissatisfaction, work dissatisfaction, organizational- culture, −behaviour, −climate, morale, motivation, commitment, involvement, professional autonomy, professional self-regulation, professional power, empowerment, conflict resolution, leadership- style –qualities, management style, managerial, conflict resolution, efficiency, organizational-, effectiveness, efficiency, productivity, performance, workflow, task performance, interprofessional relations, relation, nurse-physician, nurse-nurse, skill mix, RN mix, career mobility, professional development, learning plan, career development, clinical ladder, career ladder, job ladder, continuing education, advancement, staff experience, staff knowledge, scope of practice, professional practice, care activities, quality of health care, health care quality, quality of nursing care, nursing outcome, quality, healthcare, care, service, nursing, patient safety, patient harm, patient safety, safety
Nursing personnel
nurse, nursing staff, nurses’ aides, nursing assistant, nursing personnel, nursing workforce, nursing assistant, nursing home personnel or healthcare aide, care aide, healthcare attendant, care attendant, HCA or resident companion, geriatric aide
Surveys and questionnaires
health care surveys, −questionnaires, survey, questionnaire, reproducibility of results, validation studies, test validity, statistical validity, test reliability, statistical reliability, interrater reliability, validity, reliability, validated, reproducibility
Limitations
English, Norwegian, Danish or Swedish language

**Fig. 1:**
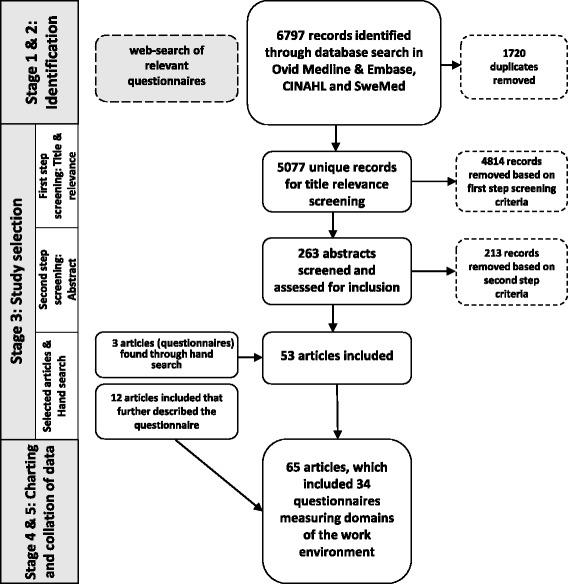
Identification, screening and inclusion/exclusion process for literature search

The reference lists of relevant articles were manually searched for additional literature. This was followed by a “snowball” procedure: when a citation in an article appeared relevant, we read the cited article. Figure 1 shows the final extensive search process illustrated in a flowchart. Our searches in electronic databases and reference lists were supplemented by targeted internet searches. Based on our familiarity with the field, we screened internet sites and publications of organizations that had previously done work in these specific or neighbouring areas, e.g. the Norwegian Association of Local and Regional Authorities (KS), the Swedish Association of Local Authorities and Regions (SALAR) and the Agency for Healthcare Research and Quality (AHRQ).

### Screening – Inclusion and exclusion

All citations from the literature search were imported into an EndNote™ library, after which duplicates and conference abstracts were manually removed. After the study selection, the citations were imported into a spreadsheet and screened for relevance and quality.

The relevance of the studies in the search was assessed using a two-step screening process. The inclusion criteria in the first step were: a) Articles in which the questionnaire in use were tested on nurses working in direct and basic nursing care in general hospitals, nursing homes or home healthcare. That is, everyday nursing care that does not require special education, and that may be performed by less qualified personnel under registered nurses’ supervision. Hence, questionnaires that could be suitable for both registered nurses, practical nurses, and nursing assistants were of main interest. In this review, the term “nurses” include the three groups. b) Self-reported survey questionnaires constructed to measure work environment characteristics that are assumed to facilitate or limit nursing practice. This refers to nursing practice outcomes related to patient and/or organizational outcomes; the latter are highly relevant in human resource management. In order to be useful in quality development, the questionnaires of interest should describe characteristics of the work environment that are amenable to change. c) Articles presenting an assessment of the measurement properties of a questionnaire were included because measurement properties are central aspects when choosing a questionnaire.

Exclusion criteria included: a) Study setting was countries with health systems and cultures that differed greatly from Norway, such as Iran or China. b) Conference abstracts, books, reports and dissertations.

The first-step screening was conducted by the first author based on the titles in the publications. If more information was needed, the abstract was browsed. In the case of uncertainty regarding inclusion, the literature was included for further screening in the second step.

In the second step, the eligibility criteria were determined on a post-hoc basis, as the two authors independently judged the relevance by screening the abstracts. Because difficulties with recruitment and turnover among nurses were identified as a global concern in the early 2000s [27], we made a choice to restrict the included literature to not older than 20 years. Review articles [28, 29] were not included, but they were examined for citations. Articles explicitly stating that a usage fee or licence was required [30] were not included. We also excluded articles describing a questionnaire that measured only one work environment characteristic such as leadership [31]. This was done because the work environment is considered a multidimensional phenomenon, and in order to have a questionnaire of a reasonable length, we excluded questionnaires that went deep into one characteristic, measuring only one dimension.

### Selected articles

Articles were included for full-text reading and charting of contents, if one author found it potentially relevant. Additional articles that presented quality assessment of the questionnaire in the article were read thoroughly in order to clarify the development, use or properties of the questionnaire. For example, if an article referred to a qualitative study that described the content development of a questionnaire or the psychometric properties of the particular questionnaire, this article was included in the appraisal of the questionnaire and charting of data.

### Data extraction and presentation

The results are mainly presented in tables as recommended by Khalil et al. [25]. Table 2 is a presentation of the characteristics of the questionnaires in order to compare and assess their relevance. If the questionnaire was used in several studies, we present these studies together.

**Table 2:** Characteristics of included studies

Author/country	Name of questionnaire	Main study object	Target group	Workplace Setting	Items/domains	Response format	Additional literature	Group/appraisal
Adams & Bond (1995) [42]/UK	The Ward Organisational Features Scales -WOFS	Environmental factors influential on the effectiveness of nursing services	Registered nurses	Hospital	105/14	4- and 5-point scales		1
Sjetne & Stavem (2006) [89]/Norway					93/12 (did not use 2 domains)			
Edvardsson et al. (2009) [40]/Sweden	Person-centred Climate Questionnaire – PCQ-S	Person-centred climate	Healthcare staff	Hospital	14/3	6-point scale		1
Bergland et al. (2012) [48]/Norway			Care staff	Nursing homes				
Edvardsson et al. (2015) [90]/Sweden			All staff on duty					
Bondevik et al. (2014) [71]/Norway	The safety attitudes questionnaire – ambulatory Norwegian version for the primary care setting – SAQ-AV	Patient safety culture	Registered nurses, medical secretaries and bioengineers, medical doctors	Out-of-hours casualty clinics & general practitioner practices	62/5	5-point scale		1
Buljac-Samardzic et al. (2016) [61]/Netherlands			Direct care employees	Nursing and residential homes				
Chou et al. (2002) [51]/UK	Measure of job satisfaction for nursing homes. Based on Traynor & Wade –MJS (1993) [91]/UK	Job satisfaction	All staff	Nursing homes	22/5	5-point scale	Traynor & Wade (1993) [91] /UK	1
Ellenbecker & Byleckie (2005) [59]/USA	Home healthcare nurse’s job sat. scale – HHNJS	Home healthcare nurse’s job satisfaction	All healthcare workers	Home healthcare	30/9	5-point scale		1
Ellenbecker et al. (2008) [92]/USA								
^a^Estabrooks et al. (2009) [36]/Canada	Alberta Context Tool – ACT	Organizational context central to evidence-based practice	Paediatric nurses	Hospitals	56–58/ 10	5-point scale		1
			Nurses (Registered nurses, licensed practical nurses)	Elder care facilities			Eldh et al. (2013) [93] /Sweden	
			Healthcare aides	Nursing homes			Estabrooks et al. (2013) [60] /Canada	
			Professional nurses	Different care settings			Squires et al. (2013) [94]/Canada	
Flint et al. (2013) [55]/Australia	Brisbane Practice Environment Measure - B-PEM	Nurses’ practice environment	Registered nurses	Hospital	26/4	5-point scale	Webster et al. (2009) [95] /Australia	1
Reid et al. (2015) [96]/Australia				Sample	28/5			
Murrells et al. (2005) [69]/UK	Instrument for job satisfaction in nursing developed for the UK	Nurses’ job satisfaction	Nurses	All settings	20/6	5-point scale		1
^a^Temkin-Greener et al. (2009) [35]/USA	Work environment and perceived work effectiveness	Nursing home work environment and perceived work effectiveness	All employees	Nursing homes	68/5	5-point scale		1
Andersson & Lindgren (2008) [53]/Sweden	Karen-personnel	Quality of care from personnel’s perspective	Registered nurses, nurse assistants	Hospital	35/6	5-point scale	Andersson & Lindgren (2013) [97] /Sweden	2
Castle (2010) [46]/USA	Nursing home certified nurse assistant job satisfaction questionnaire – NH-CNA-JSQ	Certified nurse assistant Job satisfaction	Certified nurse assistants	Nursing homes	19/7	Visual analogue rating format (10- point scale)		2
^a^de Brouwer, et al. (2014) [37]/Netherlands	Essentials of Magnetism -EOMII	Essentials of a productive nurse work environment identified by nurses practicing in Magnet hospitals	Nurses with vocational training, Bachelor degree nurses	Hospital	58/8	4-point scale	Schmalenberg & Kramer (2008) [21] /USA	2
Deilkas & Hofoss (2008) [70]/Norway	Norwegian version of the Safety Attitudes Questionnaire, Generic version (Short Form 2006) – SAQ	Patient safety culture	Physicians, nurses, physio-therapists, radiographers	Hospital	36/7	5-point scale	Sexton et al. (2006) [98] /USA, UK, New Zealand	2
From et al. (2013) [50]/Sweden	The Creative Climate Questionnaire – CCQ (Generic)	Creative climate	All care workers	Long-term care	50/10	4-point scale	Mathisen & Einarsen (2004) [99]/R	2
Ives-Erickson et al. (2015) [43]/USA	The patient care associates’ work environment scale – PCA-WES	Patient care associates’ practice environment in the acute care settings	Nurse aides	hospital	35/5	4-point scale		2
Lake (2002) [14]/USA	Practice Environment Scale of the Nursing Work Index – PES-NWI	Nurses’ practice environment	Registered nurses	Hospital	31/5	4-point scale		2
Cummings et al. (2006) [17]/Canada				Hospital				
Spence-Laschinger (2008) [66]/Canada				Hospital				
Flynn et al. (2010) [3]/USA				Nursing homes				
Gajewski et al. (2010) [100]/USA				Hospital				
Jarrin et al. (2014) [63]/USA				Home healthcare				
Lynn et al. (2009) [101]/USA	Satisfaction in Nursing Scale – SINS	Work satisfaction	Registered nurses	Hospital	55/4	5-point scale		2
Mensik (2007) [65]/USA	Dimensions of Magnetism instrument – DOM	Dimensions of Magnetism	Nurses	Home healthcare	37	Checklist −10 most important		2
Mueller & Savik (2010) [49]/USA	Nursing Practice Model Questionnaire – NPMQ	Nursing practice model	Registered nurses, licensed practical nurses, nursing assistants	Long-term care	37/5	yes/no 3-point scale		2
Slater et al. (2009) [56]/Ireland	The Nursing Context Index – NCI	Nurses practice environment (person-centred practice framework)	Registered nurses	Hospital	89/19	7-point scale.	McCormack et al. (2010) [62] /Ireland	2
				Residential settings for older people				
Tourangeau et al. (2006) [102]/Canada	McCloskey/Mueller satisfaction scale – MMSS	Nurse job satisfaction	Nurses	Hospital	23/7	5-point scale		2
Zuniga et al. (2013) [58]/Switzerland	Nursing Home Survey on Patient Safety Culture – NHSOPSC	Safety climate	Direct care nursing personnel & nursing unit supervisors	Nursing homes	42/12	5-point scale		2
Aiken & Patrician (2000) [13]/USA	Nursing Work Index-Revised – NWI-R	Nurses’ practice environment	Registered nurses	Hospital	57/4	4-point scale		3
Flynn et al. (2005) [57]/USA & New Zealand				Hospital, home care & district nursing	47/4			
Cummings et al. (2006) [17]/Canada				Hospital				
Joyce & Crooks (2007) [103]/Australia				Hospital	29/5			
Li et al. (2007) [104]/USA				Hospital	21/4			
Slater et al. (2010) [105]/Northern Ireland				Hospital	33/3			
Sjetne et al. (2010) [106]/Norway				Hospital	26/5			
Best & Thurston (2006) [107]/Canada	Index of Worklife Satisfaction – IWS	Worklife satisfaction	Public health nurses	Sample	Part A:15 Part B: 44	Paired comparisons and a 5-point scale		3
					Part B: 44	7-point scale	Zangaro & Soeken (2005) [108] /R	
Castle (2006) [64]/USA	The Hospital Survey on Patient Safety Culture –HSOPSC	Safety culture	Nurse aides	Nursing homes	42/12	5-point scale		3
Blegen et al. (2009) [109]/USA			Healthcare staff	Hospitals				
Castle et al. (2007b) [45]/USA	Nursing home nurse aide job satisfaction questionnaire – NHNA-JSQ	Nurse aide job satisfaction	Nurse aides	Nursing homes	21/7	Visual analogue rating format: 10-point scale	Castle (2007a) [110] /USA	3
Estabrooks et al. (2002) [111]/Canada	Practice Environment Index – Single factor model	Nurses’ practice environment	Registered nurses	Hospital	26/1	4-point scale		3
Cummings et al. (2006) [17]/Canada								
Fairbrother et al. (2009) [41]/Australia	Nursing Workplace Satisfaction Questionnaire – NWSQ	Job satisfaction	Nurses	Hospital	14/3	Agreement- based scale		3
Kvist et al. (2012) [112]/Finland	Kuopio University Hospital Job Satisfaction Scale – KUHJSS	Job satisfaction	Nursing staff	Hospital	37/7	5-point scale		3
Lacey et al. (2011) [113]/USA	Organizational job satisfaction – OJS	Organizational job satisfaction	Nurses	Hospital	17/NI	4-point scale		3
LaMarche & Tullai-McGuinness (2009) [47] /USA	Misener nurse practitioner job satisfaction survey – MNPJSS	Nurse practitioner job satisfaction	Nurse practitioners	Primary healthcare	44/6	6-point scale		3
	Minnesota Satisfaction Questionnaire – MSQ-SF				20/2	5-point scale		
Parmelee et al. (2009) [44]/USA	Nursing Assistants Barriers Scale – NABS	Perceived barriers to job performance	Nursing assistants	Nursing homes	30/6	NI		3
Santavirta (2003) [114]/Finland	Job content questionnaire – JCQ + parts from QPS-Nordic (Generic)	General questionnaire measuring working conditions	Teachers & nurses	Hospital	All studies used different parts/items	4- or 5-point scale		3
Larsson et al. (2013) [67]/Sweden			Home care aides, nursing assistants	Home care				
Zhang et al. (2014) [68]/USA			All employees	Nursing homes				
Tervo-Heikkinen et al. (2014) [115]/Finland	The RN Working Conditions Barometry Index form – RN-WCBI (based on NWI-R and QPS-Nordic)	Nurses’ work environment	Nurses	Sample – all settings	38 questions /207 statements	NI		3

^a^Found through reading of reference lists

*NI* No Information found in literature

*R* Review

First, we identified the study author(s) and country of development, name of the questionnaire used in the study and the main study object, i.e. the main dimension the questionnaire is designed to measure. The number of items in the questionnaire represents a total count, including questions not concerning work environment issues, but excluding sociodemographic questions. We recorded the target population in the study; this may differ from the population the questionnaire was originally developed for. The workplaces of the participants in each study and the response format used in the questionnaire are also presented in the table. In case of revisions, we extracted data from the latest version known to us.

### Brief appraisal and questionnaire content

Daudt et al. [26] suggested that scoping reviews should include some form of quality assessment for included studies. Therefore, we performed a brief appraisal by recording relevant information about psychometric properties that were presented with the questionnaire. This assessment was based on a very short customized version of the COSMIN checklist [32, 33]. For example, whether the content development was described, if reproducibility or internal consistency was tested and if it had acceptable results. The appraisal also included a global rating of scientific quality and of the overall face validity for basic nursing in long-term settings. The appraisal scores were summed, and the questionnaires were categorized in three groups according to their appraisal scores (nine with high scores in group one; thirteen in group two with medium scores; twelve in group three with low scores).

The methodological quality or risk of bias was not assessed in the included articles. This is in line with how scoping reviews are usually conducted [34].

In regard to research question 2; the questionnaire content mapping was conducted by mapping the content domains of the nine questionnaires in group 1, represented by the labels assigned to them by the authors. We decided, a priori, to build on Bae’s [7] review of working conditions. The first author did a qualitative interpretation of the domains in the questionnaires and their concurrence with Bae’s synthesised domains. This was done in order to map the domains and labels used in the questionnaires and possibly expand the range of domains already identified by Bae.

## Results

### Search and selection of literature

The first literature search was conducted in October 2015 and yielded 4305 unique articles. The update search conducted in December 2016 provided 750 new articles, after duplicates were removed. Figure 1 illustrates the search and selection. After the first relevance screening, 263 articles remained. For the final synthesis of full-text articles, 50 articles were included. Three more questionnaires measuring different dimensions of the work environment were found by screening references in the included literature [35–37]. We included 12 articles that elaborated on properties of any of the identified questionnaires; the final selection consisted of 65 articles comprising 34 questionnaires. We searched government and organization websites and found generic work environment questionnaires currently in use in many different types of services; for example the QPS-Nordic [38] and the 10-faktor [39].

### Characteristics of included questionnaires

Table 2 shows the questionnaires found in our search and is sorted first by the appraisal group and then by the author’s name.

The number of items in the questionnaires varies considerably from 14 [40, 41] to 105 [42]. The questionnaires were developed for and tested in health personnel subgroups, for example, questionnaires developed for nurse aides [43–45], practical nurses [46], nurse practitioners [47], all employees [35], all care workers [48–53], and specific versions developed for different groups of workers [36, 54]. Most questionnaires were developed and tested for registered nurses [14, 16, 37, 53, 55–57].

Most questionnaires were tested in a general hospital setting. Six questionnaires were developed specifically for use in nursing home settings [35, 44–46, 49, 58], and one [59] for home healthcare. However, four questionnaires were adapted and modified from a hospital setting for use in nursing homes [48, 51, 60, 61]. Three questionnaires were developed for use in a hospital setting but were used in the long-term care setting without modification, or with just minor changes in wording to fit the new setting [3, 62–65].

The Nursing Work Index [63, 66] and the Job Content Questionnaire [67, 68] are used in both home healthcare and nursing homes settings. In these studies, only registered nurses were included.

The most frequently used response format was a Likert-type four- or five-point scale.

There was considerable variation as to which outcomes and work environment dimensions were measured by the questionnaires. These were, for example, quality of care [53], job satisfaction [45, 46, 51, 69], safety attitudes or safety culture [58, 70, 71], creative climate [50], barriers [44], person-centred care [48], or evidence-based practice [36]. In regard to measuring only the practice environment, the most frequently used questionnaire that we found in this review is the Nursing Work Index (NWI). In our findings, the NWI is also the questionnaire that has been most revised. The nurses’ practice or work environment as the main study object was also found in a questionnaire named the Brisbane Practice Environment Measure (B-PEM) [55], which is similar to the NWI in terms of contents. Some of the questionnaires have the work environment in a specific context or setting as the main study object, such as: Work environment and perceived work effectiveness [35], Patient care associates’ practice environment in the acute care settings [43], Nurses’ practice environment (person-centred practice framework) [56], Essentials of a productive nurse work environment identified by nurses practicing in Magnet hospitals [37], and work environment as perceived by nurses [16].

The Job Content Questionnaire (JCQ) [67, 68] and the Creative Climate Questionnaire (CCQ) [50] are generic questionnaires, developed to be used in any professional group.

### Questionnaire contents

Table 3 shows the nine questionnaires in appraisal group 1, with attention paid to the questionnaire contents. The ten domains synthesized in Bae’s review [7] are presented in the top row in Table 3.

The content domains are labelled differently. For example, Bae uses the concept “supportive managers” but supposedly similar domains identified in eight out of nine questionnaires were labelled “perceptions of management” [71], “professional support” [51], “relationship with organization” [59], “leadership” [35, 36], “management support” [55] and “ward leadership” [42]. The concept of “supportive managers” was split into two domains (relationships and development) in one questionnaire [69].

The concept domain of collaborative relationships with peers was present in almost all questionnaires, but the label varied. The labels in the questionnaires were: “a climate of community” [40], “teamwork climate” [71], “team spirit” [51], “relationship with peers” [59], “informal interactions” [36], “relationships” [69] and “staff cohesion” [35] and “professional relationship amongst nurses” [42]. The collaborative relationships with the physicians’ domain were less prevalent than the relationships with peers. The label was called: “relationship between nurses and medical staff” [42] and “relationship with physician” [59]. In one questionnaire, the label was “formal interactions” [36] and included different healthcare providers, not only physicians.

The perception of busyness is also a topic in the majority of the questionnaires. These are labelled: “stress and workload” [59], “workload” [51, 55], “staff organization” [42], “organizational slack-staff”, “organizational slack–time” [36], “nature of work” [69] and “perceived work effectiveness” [35].

The domains of autonomy, participation and involvement were labelled “personal satisfaction” [51], “professional pride” and “autonomy” [59], “influence on timing of ward and patient events”, “influence on ward management” and “influence on human and financial resources” [42] and “culture” [36] in the questionnaires.

The domain of patient-centred climate was present in one questionnaire, labelled “relationship with patients” [59]. Person-centred climate was also the overall phenomenon to be measured in one questionnaire [40].

Four out of nine questionnaires contained a domain related to professional practice and education. These were labelled “professional practice” [42], “training” [51], “professional development” [55], and “education” [69]. One questionnaire had “evidence-based practice” as an overall phenomenon to be measured [36]. Professional development was not included in Bae’s [7] synthesis.

Other domains that were not present in Bae’s review were the physical surroundings and availability of resources. In the questionnaires identified in the present study, these were labelled: “ward facilities” [42], “a climate of everydayness” [40], “structural and electronic resources”, “organizational slack–space” [36] and “resources” [69].

A domain including salary, benefits and rostering was also present in the questionnaires, labelled “rostering” [55], and “salary and benefits” [59], as was patient safety, labelled “ward layout” [42], “a climate of safety” [40] and “safety climate” [51].

## Discussion

In this scoping review, we identified survey questionnaires measuring nurses’ perceptions of work environment. We have mapped the content domains included in a group of questionnaires.

The following discussion focuses first on nursing settings in general, then on the long-term care perspective.

### Overall settings

The Nursing Work Index (NWI), and modified versions of it, stand out as the most frequently used instrument for measuring the work environment of registered nurses in this review. Because the NWI was developed in the USA over 25 years ago [12], the content of the NWI may be decreasingly relevant for contemporary work settings. However, The Essentials of Magnetism (EOM) [19] process measurement tool and subsequently its revised version EOMII [20, 21] were developed with a basis in the NWI and assess more contemporary aspects of importance for a productive nursing work environment. The healthcare sector is constantly under transformation. New management structures and cost containment have been prominent features in recent years [72], as have the change from profession-centredness to patient-centredness and patient-safety focus. Taking a broader view on the work environment, the questionnaires include a varied selection of constructs and operationalisations intended to represent the work environment domains of nurses. The domains we mapped in the questionnaires are to some extent overlapping, often with little consistency in terminology. Nurses’ perceptions of their work environment may include a range of different phenomena that are not necessarily directly related to one another, but indirectly or directly comprise the environment in which the nurses work [73, 74]. Some domains are more prevalent in the reviewed questionnaires, but it is premature to conclude that these are more significant than others for measuring the work environment. Some elements may been the subject of less attention or research and therefore not measured in the questionnaires we found in this review, such as relationships with other professionals or relatives, as opposed to relationships with physicians and peers, which are the commonly measured domains.

Our findings illustrate the importance of clarifying and defining the outcome one intends to measure. When measuring a broad construct, in our case “nursing work environment”, the subdomains of relevance for the target population and in the specific context need to be defined [33, 75]. In our review, several questionnaires seem to measure the same or overlapping domains, but under different labels and uniquely operationalised. For example, the domain “autonomy” is a common work environment domain included in work environment surveys. It has been argued that the concept of autonomy can be theoretically differentiated into two discrete concepts – autonomy related to the nurses’ clinical practice and autonomy in relation to work [76]. When measuring a domain such as “autonomy”, one needs to clarify the theoretical construct, and be aware that a measure of a construct in one questionnaire may not be used interchangeably with another construct of the concept in a different questionnaire. This means that a theoretical consideration of how nurses’ work environments are conceived needs to be made, and made explicit, when choosing among questionnaires and in the design of a study [7].

### Long-term care settings

Based on a review of national frameworks of long-term care quality policy documents and analytic frameworks in the academic literature, the Organization for Economic Co-operation and Development (OECD) stressed three aspects as generally accepted and critical underpinnings of the quality of long-term care: patient-centredness, care co-ordination, and safety effectiveness [77]. The dimension of patient-centred climate was not a common dimension in the first group of the reviewed questionnaires. One questionnaire measured person-centred climate as a sole topic [40]. Patient-centredness has become a healthcare quality hallmark and may represent something slightly different in long-term care settings compared to acute care settings. Nurses in long-term care deem social relationships with residents to be an important factor of their work environment and in their assessment of quality of care and their intent to remain in their work [78], as well as a motivating factor that is important for their job satisfaction [79]. This is supported by previous research, which found that residents in nursing homes find the relationships with nurses to be an important factor in their wellbeing and in high-quality care [80, 81].

The dimension of collaborative relations with peers was an aspect in almost all nine questionnaires. Researchers found that team collaboration and performance are associated with higher levels of quality of care and functional outcomes among residents in nursing homes [82, 83]. Nursing home staff’s perceptions of better team climate were related to better-perceived quality of care in a recent study, and the researchers concluded that team climate was an important factor to consider when trying to improve quality of care [84]. It is also argued that there is a possible association between improved teamwork and reduced work stressors and less care left undone [85].

The last aspect OECD stresses as important to quality of long-term care is “safety” [77]. We found this domain in the questionnaires. Nursing care is provided to patients in complex care environments that can generate errors and cause harm. Patient safety is also considered an indicator of high-quality nursing care. This can be seen as related to nurses’ direct roles in integrating care, detecting possible errors and preventing harm and adverse events [86]. A failure in fulfilling these roles may result in errors in patient care as well as adverse events. The aspect of safety climate may therefore be among the most important factors when measuring the work environment in long-term care settings.

An important phenomenon that was absent in the questionnaires was the relationship with relatives. As the residents in nursing homes need more complex care, in cases of, for example, dementia, the relatives’ role as “spokespersons” will be crucial for patient-centredness [87]. The relatives’ role may grow in importance and become more central among the prerequisites that facilitate good nursing practice.

### Limitations

The literature about the work environment is large and complex, with a wide variety of constructs and operationalisations to represent the nursing work environment, often with little consistency in the use of terminology [74]. This means that there may be terms that pertain to the work environment domains that we did not include in the search. As a result, questionnaires may have been neglected by the procedures we followed. The first screening was done by reading the title, and browsing the abstract in case of uncertainty. The precision of this procedure is entirely dependent on the terminology used in the titles and the abstracts. There is a risk that relevant articles may have been overlooked for this reason. We directed our search to factors that are amenable to change and that pertain to the aspects of professional nursing practice. Consequently, questionnaires may have been filtered out because we found that the main content concerned personal or psychosocial characteristics, while, in fact, a part of the questionnaire may have fitted our aim. Conversely, some of the questionnaires included in the review may have domains relating to psychosocial or personal aspects. There may also be questionnaires used by governments and organizations that our internet searches did not find. The search was also conducted with terms including measurement properties (included in Table 1), i.e. articles that did not present measurement properties could have been sorted out, even though they may fit our criteria.

**Table 3:** Content in group one questionnaires

Author	Name of questionnaire	Domains in questionnaire
Bae (2011) [7]	Literature review of nurse working conditions and patient outcomes	Autonomy, philosophy emphasizing quality of clinical care, nurse participation, supportive managers, collaborative relationships with physicians or peers, staffing and resource adequacy, decentralized involvement in unit decision-making, patient-centred climate and busyness.
Adams & Bond (1995) [42]	The Ward Organisational Features Scales –WOFS	Ward facilities, staff organization, ward layout, professional practice, hierarchical practice, ward leadership, relationship between nurses and medical staff, professional relationship amongst nurses, influence on timing of ward and patient events, influence on ward management, influence on human and financial resources, job satisfaction
Edvardsson et al. (2009) [40]	Person-centred climate questionnaire – PCQ-S	A climate of safety, a climate of everydayness and a climate of community
Bondevik et al. (2014) [71]	The safety attitudes questionnaire ambulatory version – SAQ-AV	Teamwork climate, safety climate, job satisfaction, working conditions and perceptions of management
Chou et al. (2002) [51]	Measure of job satisfaction for nursing homes	Personal satisfaction, workload, professional support, team spirit and training
Ellenbecker and Byleckie (2005) [59]	Home healthcare nurse’s job sat. scale – HHNJS-(revised 2008)	Relationship with peers, relationship with organization, relationship with physician, salary and benefits, stress and workload, relationship with patients, professional pride, autonomy and control
Estabrooks et al. (2009) [36]	Alberta Context tool – ACT	Leadership, culture, evaluation, social capital, formal interactions, informal interactions, structural and electronic resources, organizational slack–staff, organizational slack–space, and organizational slack–time
Flint et al. (2010) [55]	Brisbane Practice Environment Measure – B-PEM	Professional development, management support, rostering, out of depth and workload
Murrells et al. (2005) [69]	Instrument for job satisfaction in nursing developed for the UK	Nature of work, development, relationships, education, work-life Interface and resources
Temkin-Greener et al. (2009) [35]	Work environment and perceived work effectiveness	Leadership, communication & coordination, conflict management, staff cohesion and perceived work effectiveness

The appraisal in this review should not be seen as a complete quality assessment, rather an appraisal of the questionnaires’ “fit” to a generic group of nursing personnel and setting.

Our results may also be influenced by some degree of dissemination bias [88], because questionnaire developers may be less willing to publish results that are unfavourable in terms of the psychometric properties of a questionnaire.

## Conclusions

This scoping review identified a large number of heterogeneous work environment questionnaires. The findings from this review enhance the understanding how “work environment” can be measured with self-reported questionnaires by providing an overview of existing questionnaires and domains. The categorization of results in Tables 2 and 3 offers clarity in synthesis and in the presentation of results, providing information that is of importance when choosing a questionnaire. In future research, it is important to further investigate and clarify which work environment dimensions are the most relevant to measure for nurses in the practice setting in question.

## Additional file

Additional file 1: Complete list of search terms. Search terms used for search in the following databases: Embase (1974-) Ovid MEDLINE(R) In-Process & Other Non-Indexed Citations, Ovid MEDLINE(R) Daily, Ovid MEDLINE(R) and Ovid OLDMEDLINE(R) (1946 -); PsycINFO (1806-); CINAHL and SweMed +. (DOCX 21 kb)

## Abbreviations

ACT: Alberta Context Tool
AHRQ: Agency for Healthcare Research and Quality
B-PEM: Brisbane Practice Environment Measure
CCQ: Creative Climate Questionnaire
CCQ: The Creative Climate Questionnaire
EOM: Essentials of Magnetism tool Norwegian
HHNJS: Home healthcare nurse’s job satisfaction scale
HSOPSC: The Hospital Survey on Patient Safety Culture
IWS: Index of Worklife Satisfaction
JCQ: The Job Content Questionnaire
KS: Association of Local and Regional Authorities (Norway)
KUHJSS: Kuopio University Hospital Job Satisfaction Scale
MJS: Measure of job satisfaction for nursing homes
MMSS: McCloskey/Mueller satisfaction scale
MNPJSS: Misener nurse practitioner job satisfaction survey
MSQ-SF: Minnesota Satisfaction Questionnaire
NABS: Nursing Assistants Barriers Scale
NCI: The Nursing Context Index
NH-CNA-JSQ: Nursing home certified nurse assistant job satisfaction questionnaire
NHNA-JSQ: Nursing home nurse aide job satisfaction questionnaire
NHSOPSC: Nursing Home Survey on Patient Safety Culture
NI: No Information found in literature
NPMQ: Nursing Practice Model Questionnaire
NWI: Nursing Work Index
NWI-R: Nursing Work Index-Revised
NWSQ: Nursing Workplace Satisfaction Questionnaire
OECD: Organization for Economic Co-operation and Development
OJS: Organizational job satisfaction
PCA-WES: The patient care associates’ work environment scale
PCQ-S: Person-centred Climate Questionnaire
PES-NWI: Practice Environment Scale of the Nursing Work Index
R: Review
RN-WCBI: The RN Working Conditions Barometry Index form
SALAR: The Swedish Association of Local Authorities and Regions
SAQ: Safety Attitudes Questionnaire, Generic version
SAQ-AV: The safety attitudes questionnaire ambulatory version
SINS: Satisfaction in Nursing Scale
UK: United Kingdom
USA: United States of America
WOFS: The Ward Organisational Features Scales
