# Supplementary material for: Measuring nurses’ perception of work environment: a scoping review of questionnaires
Source: BMC Nurs. 2017 Nov 21;16:66. doi: 10.1186/s12912-017-0256-9 (PMC5697362; doi:10.1186/s12912-017-0256-9)
Supplement: Additional file 1: — Complete list of search terms. Search terms used for search in the following databases: Embase (1974-) Ovid MEDLINE(R) In-Process & Other Non-Indexed Citations, Ovid MEDLINE(R) Daily, Ovid MEDLINE(R) and Ovid OLDMEDLINE(R) (1946 -); PsycINFO (1806-); CINAHL and SweMed +. (DOCX 21 kb) [file 12912_2017_256_MOESM1_ESM.docx]

**Embase (1974-) Ovid MEDLINE(R) In-Process & Other Non-Indexed Citations, Ovid MEDLINE(R) Daily, Ovid MEDLINE(R) and Ovid OLDMEDLINE(R) (1946-), PsycINFO (1806-)**

Date of search: 15.10.2015 + 20.12.2016

| **#** | **Searches** |
| --- | --- |
| 1 | occupational health/ |
| 2 | (occupational health or occupational safety or employee health or employee safety or occupational injur*).tw,kw. |
| 3 | working conditions/ or (work* condition* or practice environment or work* environment).tw,kw. |
| 4 | workload/ or work load/ |
| 5 | (workload or work-load or overwork* or work stressor* or nurse-patient-ratio*).tw,kw. |
| 6 | (((missed or omitted or rationing) adj2 care) or nursing left undone or care left undone).tw,kw. |
| 7 | work schedule tolerance/ or workday shifts/ |
| 8 | (work shift* or rotating shift* or workday shift* or work schedule* or work rest cycle*).tw,kw. |
| 9 | personnel turnover/ or employee turnover/ |
| 10 | (turnover or intention-to-leave or vacanc*).tw,kw. |
| 11 | "personnel staffing and scheduling"/ or work scheduling/ |
| 12 | (staffing or manpower).tw,kw. |
| 13 | burnout, professional/ or occupational stress/ |
| 14 | (burnout or exhaust* or distress or occupational stress*).tw,kw. |
| 15 | absenteeism/ or employee absenteeism/ |
| 16 | (absenteeism or sickleave or sick leave or sick* absence or sick* rate* or sick* day* or illness day*).tw,kw. |
| 17 | job satisfaction/ |
| 18 | (job satisfaction or work satisfaction or employ* satisfaction or career satisfaction).tw,kw. |
| 19 | employee grievances/ |
| 20 | (employee grievance* or personnel grievance* or nurs* grievance* or work* grievance* or staff grievance* or job dissatisfaction or work dissatisfaction).tw,kw. |
| 21 | organizational culture/ or organizational behavior/ |
| 22 | (organi?ational culture or organi?ational climate or organi?ational behavior?r*).tw,kw. |
| 23 | morale/ |
| 24 | (morale or motivation or commitment or involvement).tw,kw. |
| 25 | professional autonomy/ |
| 26 | (professional autonomy or professional self-regulation or professional power or empowerment).tw,kw. |
| 27 | conflict resolution/ or leadership style/ or leadership qualities/ or (leadership style* or management style* or managerial style* or leadership qualit* or conflict resolution).tw,kw. |
| 28 | efficiency, organizational/ or organizational effectiveness/ |
| 29 | (organi?ational efficiency or organi?ational productivity or organi?ational effectiveness or organi?ational performance).tw,kw. |
| 30 | (work flow or workflow or task performance).tw,kw. |
| 31 | exp interprofessional relations/ |
| 32 | (interprofessional relation* or nurse-physician or nurse-nurse or skill mix or RN mix).tw,kw. |
| 33 | career mobility/ |
| 34 | (professional development or learning plan* or career development or clinical ladder* or career ladder* or clinical ladder* or job ladder* or continuing education or advancement or staff experience* or staff knowledge).tw,kw. |
| 35 | ((nurs* adj3 "scope of practice") or professional practice or (nurs* adj3 care activit*)).tw,kw. |
| 36 | or/1-35 [arbeidsvilkår] |
| 37 | quality of health care/ or health care quality/ or quality of nursing care/ or nursing outcome/ or (quality adj4 (healthcare or care or service* or nursing)).tw,kw. |
| 38 | patient safety/ or patient harm/ or (patient safety or (safety adj3 quality)).tw,kw. |
| 39 | 37 or 38 [kvalitet & pasientsikkerhet] |
| 40 | exp nurses/ or exp nursing staff/ or nurses' aides/ or nursing assistant/ or (nurse* or nursing staff or nursing personnel or nursing workforce or nursing assistant* or nursing home personnel or healthcare aide* or care aide* or healthcare attendant* or care attendant* or HCA or resident companion* or geriatric aide*).tw,kw. [sykepleiere] |
| 41 | health care surveys/ or questionnaires/ or (survey* or questionnaire*).tw. [surveys] |
| 42 | exp reproducibility of results/ or validation studies.pt. use pmoz |
| 43 | exp reliability/ or exp validity/ use oemezd |
| 44 | test validity/ or statistical validity/ or test reliability/ or statistical reliability/ or interrater reliability/ use psyh |
| 45 | (validity or reliab* or validat* or reproducib*).tw. |
| 46 | or/42-45 |
| 47 | 36 and 40 and 41 and 46 [validerte spørreskjema sykepleieres arbeidsvilkår] |
| 48 | 39 and 40 and 41 and 46 [validerte spørreskjema sykepleiere kvalitet & pasientsikkerhet] |
| 49 | remove duplicates from 47 |
| 50 | remove duplicates from 48 |
| 51 | 49 or 50 |
| 52 | limit 51 to (danish or english or norwegian or swedish) |

**CINAHL**

Date of search: 15.10.2015 + 20.12.2016

| S29 | S27 OR S28 | 1,492 |
| --- | --- | --- |
| S28 | S26 AND (S19 OR S20) AND (S21 OR S22) AND (S23 OR S24)  Limiters - Exclude MEDLINE records; Language: Danish, English, Norwegian, Swedish | 224 |
| S27 | S25 AND (S19 OR S20) AND (S21 OR S22) AND (S23 OR S24)  Limiters - Exclude MEDLINE records; Language: Danish, English, Norwegian, Swedish | 1,464 |
| S26 | S15 OR S16 OR S17 OR S18 | 95,380 |
| S25 | S4 OR S5 OR S6 OR S7 OR S8 OR S9 OR S10 OR S11 OR S12 OR S13 OR S14 | 460,481 |
| S24 | TI (validity or reliab* or validat* or reproducib*) OR AB (validity or reliab* or validat* or reproducib*) | 83,696 |
| S23 | (MH "Reliability and Validity+") | 124,169 |
| S22 | TI ( survey* or questionnaire* ) OR AB ( survey* or questionnaire* ) | 171,335 |
| S21 | (MH "Surveys") OR (MH "Questionnaires+") | 243,787 |
| S20 | TI (nurse* or nursing-staff or nursing-personnel or nursing-workforce or nursing-assistant* or nursing-home-personnel or healthcare-aide* or care-aide* or healthcare-attendant* or care-attendant* or HCA or resident-companion* or geriatric-aide*) OR AB (nurse* or nursing-staff or nursing-personnel or nursing-workforce or nursing-assistant* or nursing-home-personnel or healthcare-aide* or care-aide* or healthcare-attendant* or care-attendant* or HCA or resident-companion* or geriatric-aide*) | 225,079 |
| S19 | (MH "Nurses+") OR (MH "Nursing Assistants") OR (MH "Nursing Home Personnel") OR (MH "Nursing Staff, Hospital") OR (MH "Nurse Midwives") | 163,057 |
| S18 | TI ( patient-safety or (safety W2 quality) ) OR AB ( patient-safety or (safety W2 quality) ) | 10,748 |
| S17 | (MH "Patient Safety") OR (MH "Hand Off (Patient Safety)+") | 30,164 |
| S16 | TI ( (quality W3 (healthcare or care or service* or nursing)) ) OR AB ( (quality W3 (healthcare or care or service* or nursing)) ) | 26,579 |
| S15 | (MH "Quality of Health Care") OR (MH "Quality of Nursing Care") | 48,561 |
| S14 | (MH "Staff Development") OR TI ( staff-development or professional-development or learning-plan* or continuing-education or staff-experience* or staff-knowledge ) OR AB ( staff-development or professional-development or learning-plan* or continuing-education or staff-experience* or staff-knowledge ) | 33,456 |
| S13 | (MH "Personnel Management") OR (MH "Management Styles") OR (MH "Conflict Management") OR TI ( leadership-style* or management-style* or managerial-style* or leadership-qualit* or conflict-resolution or conflict-management ) OR AB ( leadership-style* or management-style* or managerial-style* or leadership-qualit* or conflict-resolution or conflict-management ) | 9,713 |
| S12 | (MH "Career Mobility+") OR TI (career-mobility OR career-development OR clinical-ladder* OR career-ladder* OR clinical-ladder* OR job-ladder* OR advancement) OR AB (career-mobility OR career-development OR clinical-ladder* OR career-ladder* OR clinical-ladder* OR job-ladder* OR advancement) | 9,753 |
| S11 | (MH "Interprofessional Relations+") OR (MH "Intraprofessional Relations") OR TI (interprofessional-relation* or intraprofessional-relation* or nurse-physician or nurse-nurse) OR AB (interprofessional-relation* or intraprofessional-relation* or nurse-physician or nurse-nurse) | 24,303 |
| S10 | (MH "Professional Autonomy") OR (MH "Delegation of Authority") OR (MH "Empowerment") OR TI (professional-autonomy OR professional-self-regulation OR professional-power OR empowerment) OR AB (professional-autonomy OR professional-self-regulation OR professional-power OR empowerment) | 15,043 |
| S9 | (MH "Morale") OR TI (morale OR motivation OR commitment OR involvement) AND AB (morale OR motivation OR commitment OR involvement) | 4,454 |
| S8 | (MH "Organizational Culture") OR (MH "Organizational Efficiency+") OR TI (organi?ational-efficiency or organi?ational-productivity or organi?ational-effectiveness OR organi?ational-performance OR organi?ational culture or organi?ational climate or organi?ational behavior?r* or work-flow or workflow or task-performance) OR AB (organi?ational-efficiency or organi?ational-productivity or organi?ational-effectiveness OR organi?ational-performance OR organi?ational culture or organi?ational climate or organi?ational behavior?r* or work-flow or workflow or task-performance) | 34,088 |
| S7 | (MH "Job Satisfaction+") OR TI (job-satisfaction or work-satisfaction or staff-satisfaction or nurse-satisfaction or personnel-satisfaction or occupational-stress* or burnout or exhaust* or distress) OR AB (job-satisfaction or work-satisfaction or staff-satisfaction or nurse-satisfaction or personnel-satisfaction or occupational-stress* or burnout or exhaust* or distress) | 47,357 |
| S6 | (MH "Sick Leave") OR TI (absenteeism or sickleave or sick-leave or sick*-absence or sick*-rate* or sick*-day* or illness-day*) OR AB (absenteeism or sickleave or sick leave or sick* absence or sick* rate* or sick* day* or illness day*) | 5,414 |
| S5 | (MH "Employee Grievances") OR (MH "Personnel Turnover") OR TI (((personnel OR nurse* OR employee OR work* OR staff) W0 grievance*) or ((job or work or staff or nurse or personnel W0 dissatisfaction) or turnover or intention-to-leave or vacanc*) OR AB (((personnel OR nurse* OR employee OR work* OR staff) W0 grievance*) or ((job or work or staff or nurse or personnel W0 dissatisfaction) or turnover or intention-to-leave or vacanc*) | 362,123 |
| S4 | (MH "Occupational Health+") OR (MH "Occupational Stress+") OR (MH "Work Environment") OR TI (occupational-health or occupational-safety or employee-health or employee-safety or occupational-injur* or working-condition* or work*-environment* or practice-environment* OR occupational-stress* OR burnout OR exhaust*) OR AB (occupational-health or occupational-safety or employee-health or employee-safety or occupational-injur* or working-condition* or work*-environment* or practice-environment* OR occupational-stress* OR burnout OR exhaust*) | 56,774 |
| S3 | (MH "Scope of Nursing Practice") OR (MH "Professional Practice") OR TI ( professional-practice OR (nurs* W2 scope-of-practice) ) OR AB ( professional-practice OR (nurs* W2 scope-of-practice) ) | 13,968 |
| S2 | (MH "Shiftwork") OR TI ( work-shift* or rotating-shift* or workday-shift* or work-schedule* or work-rest-cycle* ) OR AB ( work-shift* or rotating-shift* or workday-shift* or work-schedule* or work-rest-cycle* ) | 2,522 |
| S1 | (MH "Skill Mix+") OR (MH "Understaffing") OR (MH "Nursing Manpower+") OR (MH "Nurse-Patient Ratio") OR (MH "Workload") OR TI ( workload or work-load or overwork* or work-stressor* or nurse-patient-ratio* or staffing or manpower or ((missed or omitted or omission or rationing) W1 care) or ((nursing or care) W0 left-undone) ) OR AB ( workload or work-load or overwork* or work-stressor* or nurse-patient-ratio* or staffing or manpower or ((missed or omitted or omission or rationing) W1 care) or ((nursing or care) W0 left-undone) ) | 185,884 |

**SveMed+**

Date of search: 15.10.2015 + 20.12.2016

| 1 | noexp:"occupational health" |
| --- | --- |
| 3 | noexp:"workload" |
| 4 | noexp:"work schedule tolerance" |
| 5 | noexp:"personnel turnover" |
| 7 | noexp:"Personnel Staffing and Scheduling" |
| 8 | noexp:"Burnout, Professional" |
| 9 | noexp:"Absenteeism" |
| 10 | noexp:"Job Satisfaction" |
| 11 | noexp:"employee grievances" |
| 12 | noexp:"organizational culture" |
| 13 | noexp:"Morale" |
| 14 | noexp:"Professional Autonomy" |
| 15 | noexp:"Efficiency, Organizational" |
| 16 | exp:"Interprofessional Relations" |
| 17 | noexp:"Career Mobility" |
| 18 | noexp:"Quality of Health Care" |
| 19 | noexp:"patient safety" |
| 20 | noexp:"Patient Harm" |
| 21 | exp:"Nurses" |
| 22 | noexp:"Nurses%27 Aides" |
| 23 | exp:"Nursing Staff" |
| 24 | noexp:"Health Care Surveys" |
| 25 | noexp:"Questionnaires" |
| 26 | #1 OR #3 OR #4 OR #5 OR #7 OR #8 OR #9 OR #10 OR #11 OR #12 OR #13 OR #14 OR #15 OR #16 OR #17 |
| 27 | #18 OR #19 OR #20 |
| 28 | #21 OR #22 OR #23 |
| 29 | #24 OR #25 |
| 30 | (#26 OR #27) AND #28 AND #29 |
